# Efficacy and Safety of Low-dose Oral Prednisolone for Patients with Refractory Hunner-type Interstitial Cystitis

**DOI:** 10.1016/j.euros.2023.07.006

**Published:** 2023-08-26

**Authors:** Yoshiyuki Akiyama, Aya Niimi, Akira Nomiya, Satoru Taguchi, Yuta Yamada, Yusuke Sato, Daisuke Yamada, Daichi Maeda, Tetsuo Ushiku, Haruki Kume, Yukio Homma

**Affiliations:** a Department of Urology, Graduate School of Medicine, The University of Tokyo, Tokyo, Japan; b Department of Urology, New Tokyo Hospital, Chiba, Japan; c Department of Urology, Kanto Rosai Hospital, Kanagawa, Japan; d Department of Molecular and Cellular Pathology, Graduate School of Medical Sciences, Kanazawa University, Kanazawa, Japan; e Department of Pathology, Graduate School of Medicine, The University of Tokyo, Tokyo, Japan; f Department of Interstitial Cystitis Medicine, Kyorin University School of Medicine, Tokyo, Japan

**Keywords:** Interstitial cystitis, Bladder pain syndrome, Corticosteroid, Hunner lesion, Prednisone, Prednisolone

## Abstract

**Background:**

Hunner-type interstitial cystitis (HIC) is an immunological, chronic inflammatory disease. The efficacy of corticosteroid as a treatment for HIC is unclear.

**Objective:**

To assess the efficacy and safety of low-dose oral prednisolone (PSL) treatment for patients with refractory HIC.

**Design, setting, and participants:**

This retrospective observational study reviewed the clinical outcomes of 31 patients with refractory HIC who received oral PSL daily (initial dose, 5.0 or 7.5 mg) for at least 12 mo between 2016 and 2023. The dose was tapered to the minimum that maintained symptom relief during follow-up.

**Outcome measurements and statistical analysis:**

Treatment outcomes were evaluated using a seven-graded global response assessment (scores ≥+2, moderately or markedly improved, were defined as treatment response), O’Leary and Sant symptom and problem indices (OSSI/OSPI), overactive bladder symptom score (OABSS), an 11-point pain intensity numerical rating scale, a quality of life (QOL) score, and frequency-volume chart variables. Related complications were also documented.

**Results and limitations:**

The mean follow-up period was 20.1 ± 14.6 mo. The overall response rates at 1, 3, 6, 9, and 12 mo at doses of 6.7, 6.7, 5.2, 4.0, and 3.0 mg were 38.7%, 48.4%, 54.8%, 61.3%, and 64.5%, respectively. Compared with baseline, OSSI/OSPI and pain intensity improved significantly from 1 mo after PSL induction. The OABSS, QOL score, urinary frequency, and voided volume improved significantly from 9 mo after PSL induction. No patients discontinued treatment due to adverse events, although hypertension and glucose intolerance occurred in two patients, but these were resolved by temporal medications.

**Conclusions:**

This study showed that low-dose oral PSL significantly improves bladder pain, urinary symptoms, and QOL in patients with HIC, without serious adverse events. Further prospective evaluation is warranted to verify the potential efficacy and safety of low-dose PSL for HIC.

**Patient summary:**

This retrospective observational study reviewed the clinical outcomes of 31 patients suffering from refractory Hunner-type interstitial cystitis treated with low-dose oral prednisolone. Low-dose prednisolone improved bladder pain, urinary symptoms, and quality of life significantly, without serious adverse events. The response rate of 64.5% at 12 mo was comparable with the rates reported in previous studies that used higher doses of prednisolone. This study provides a rationale for further prospective evaluation of low-dose prednisolone for this intractable disease.

## Take Home Message

This retrospective observational study showed that low-dose prednisolone improved bladder pain, urinary symptoms, and quality of life significantly, without serious adverse events, in 31 patients with refractory Hunner-type interstitial cystitis. The data provide a rationale for further prospective evaluation of low-dose prednisolone as a treatment for this intractable disease.

## 1 Introduction

Interstitial cystitis/bladder pain syndrome (IC/BPS) is an intractable, devastating urological disorder of unknown etiology, commonly characterized by persistent pelvic/urethral pain in conjunction with lower urinary tract symptoms such as urinary frequency and urgency [1], [2]. IC/BPS is subdivided into two subtypes, Hunner-type IC (HIC) and BPS, based on the presence or absence of Hunner lesions [1]. Past studies show that HIC is a distinct chronic inflammatory disease characterized histologically by lymphoplasmacytic infiltration, epithelial denudation, stromal edema, and hyperemia, while BPS is a noninflammatory disease with little evidence of bladder pathology and potentially associated with systemic neurophysiological dysregulation [3], [4], [5]. Growing evidence suggests that HIC is an immune-mediated inflammatory disease, possibly autoimmune in nature [[4], [6], [7], [29]]. Patients with HIC have a high incidence of autoimmunity and high titers of autoantibodies in the serum and bladder [8], [9]. In addition, systemic autoimmune diseases, including Sjogren’s syndrome and systemic lupus erythematosus (SLE), are frequently accompanied by irritable bladder disorders that present with HIC-like symptoms [10]. Patients with HIC show an increased prevalence of comorbid systemic autoimmune disorders [7]. Furthermore, female preponderance and precedent microbiome infection, typical epidemiological features of systemic autoimmune disorders, are also well-known features of patients with HIC [7]. Recently, we found clonal expansion of infiltrating B cells, accompanied by increased C-X-C motif chemokine receptor (CXCR) 3 expression, in the HIC bladder [5], [11]. We also observed that the HIC bladder shows gene expression patterns similar to those observed in autoimmune diseases, as well as enriched biological pathways related to systemic autoimmune diseases such as SLE, rheumatoid arthritis, autoimmune thyroid disease, and type-1 diabetes mellitus [6]. Taken together, these findings strongly suggest a possible autoimmune mechanism underlying the pathophysiology of HIC. In this context, it is of interest that prior studies demonstrate that systemic administration of immunomodulatory drugs, such as prednisolone (PSL, a corticosteroid), cyclosporine A (an immunosuppressive drug), and certolizumab pegol (an antitumor necrosis factor antibody), improves symptoms significantly in patients with HIC [12], [13], [14], [15], [16], [17]. However, the current clinical guidelines for IC/BPS do not recommend proactive use of these immunomodulatory agents due to concerns about adverse systemic effects that may outweigh their clinical benefits [1], [2]. In the present study, we treated patients with refractory HIC using low-dose PSL, starting at a dose of <7.5 mg/body, and found that it improved symptoms without significant adverse events such as glucose intolerance, increase in blood pressure, glaucoma, or glucocorticoid-induced osteoporosis [18], [19]. Herein, we report that long-term, low-dose, oral PSL significantly ameliorates irritable pain and voiding symptoms, increases bladder capacity, and improves quality of life (QOL) in patients with refractory HIC without any serious adverse events, achieving treatment success rates comparable with those of past immunomodulatory therapies of higher doses of PSL or cyclosporine A.

## 2 Patients and methods

### 2.1 Ethics statement

The present study, including the use of an opt-out methodology to obtain informed consent, was approved by the Institutional Review Board of the University of Tokyo (approval no. 3124). Patients were informed about the study using generally accessible contact information, and written informed consent was obtained from patients who chose to take part. All procedures followed appropriate guidelines.

### 2.2 Patients

This study is a retrospective observational study of a prospectively maintained database of patients with refractory HIC treated with low-dose oral PSL between 2016 and 2023. A diagnosis of HIC was based on the East Asian clinical guidelines and the International Society for the Study of BPS criteria [1], [20]. All patients had been treated at least once with electrocautery of Hunner lesions with concomitant bladder hydrodistension in conjunction with other conservative therapies, including intravesical instillation of dimethyl sulfoxide (DMSO). PSL treatment was considered when a patient’s pain relapsed after the last endoscopic surgery with or without subsequent DMSO treatment, and could not be managed by a combination of analgesic agents. Details of the eligibility criteria for PSL treatment are described in Supplementary Table 1. Histology of bladder mucosal biopsies obtained at the time of surgery was assessed to check whether the bladder manifested histological features characteristic of HIC, such as a predominant lymphoplasmacytic infiltration outnumbering granulocyte infiltration, stromal edema and fibrosis, and epithelial denudation [3], [5]. PSL was not given when the bladder did not show these histological characteristics consistent with HIC (Supplementary Table 1). Diagnosis, endoscopic surgery, and PSL treatment were performed consistently by a single urologist (Y.A.).

### 2.3 Steroid therapy

Eligible patients were administered oral PSL starting at a dose of 5.0 or 7.5 mg/body daily. When patients showed continual symptom improvement for over 3 mo, the dose was tapered gradually at every visit, and the PSL administration was eventually terminated. If symptoms exacerbated at a reduced dose, the dose was set back to the previous level. Prophylactic bisphosphonate or denosumab was administered concurrently with PSL to the required patients according to the guidelines for the management and treatment of glucocorticoid-induced osteoporosis published by the Japanese Society for Bone and Mineral Research [19]. Meanwhile, a low dose of PSL (<7.5 mg) allowed the omission of a prescription for prophylactic antibacterial agents during PSL treatment. Patients attended follow-up visits every month for the first 6 mo after induction, and every 3 mo thereafter. No other treatments were given in parallel during PSL treatment, while additional use of analgesic agents, such as opioids, acetaminophen, pregabalin, or nonsteroidal anti-inflammatory drugs (NSAIDs), was allowed.

### 2.4 Outcome assessment

Treatment outcomes were evaluated at 1, 3, 6, 9, and 12 mo after PSL induction. Despite the mean follow-up period of over a year (20.1 ± 14.6 mo), the clinical outcomes were analyzed for up to 12 mo because the number of patients who continued PSL treatment decreased gradually after 12 mo due to symptom remission or treatment failure. Treatment response was evaluated using a global response assessment (GRA) questionnaire, which is a seven-point symmetric scale that evaluates changes in the overall conditions of patients from the start of treatment: markedly improved (+3), moderately improved (+2), slightly improved (+1), no change (0), slightly worse (–1), moderately worse (–2), and markedly worse (–3). Patients who rated the treatment efficacy as moderately improved (+2) or markedly improved (+3) in the GRA scale were considered as responders. Treatment failure was defined as the continual observation of GRA scores of ≤0 (no change or worse) for over 3 mo with a treatment dose of PSL. When patients experienced treatment failure, PSL was discontinued after appropriate dose tapering. Patient symptoms were evaluated using the IC/BPS symptom scores measured by O’Leary and Sant symptom index (OSSI) and problem index (OSPI); 11-point numerical rating of pain intensity, with 0 indicating no pain and 10 indicating the worst pain ever; a seven-grade QOL scale derived from the International Prostate Symptom Score, with 0 indicating excellent and 6 indicating terrible; and the overactive bladder symptom score (OABSS). Daytime and nocturnal urinary frequency, and maximum and average voided volume were also documented. Demographic information, including age at the commencement of PSL treatment, duration of illness, previous treatments, and maximum bladder capacity measured at the last session of bladder hydrodistension at a pressure of 80 cmH_2_O under general anesthesia, was also documented.

### 2.5 Safety assessment

Adverse events and side effects occurring during PSL treatment were monitored carefully. At every follow-up visit, all patients underwent blood and urine analyses, and were checked for physical manifestations, vital signs, blood counts and electrolytes, renal and hepatobiliary function, serum C-reactive protein levels, hyperglycemia, urinary tract infection, and hypertension.

### 2.6 Statistical analysis

Multiple comparisons between the symptom parameters at each follow-up visit and baseline were evaluated using the Friedman test, followed by a two-tailed, pairwise comparison with Wilcoxon signed-rank test with post hoc Bonferroni correction. A logistic regression analysis of baseline characteristics was applied to identify factors predictive of treatment response at 12 mo. A *p* value of <0.05 (or 0.0033 for multiple comparisons) was considered statistically significant. All statistical analyses were calculated by JMP, version 14 (SAS Institute, Cary, NC, USA). Data are expressed as the mean ± standard deviation.

## 3 Results

### 3.1 Patients

A total of 31 patients (mean age, 66.3 ± 12.1 yr) with HIC (including 25 females) were included in the study. Demographics of the study participants are shown in Table 1. Patients had undergone a mean number of 2.7 ± 1.1 sessions of transurethral electrocautery of Hunner lesions with bladder hydrodistension, and three of them had also undergone a single course (six instillations for 12 wk) of intravesical DMSO therapy after surgery. Among the previous medicines listed in Table 1, NSAIDs, acetaminophen, opioids, and pregabalin were allowed during PSL treatment.

**Table 1:** Demographics of the study participants

	Male	Female	Total
No. of patients	6	25	31
Mean age (yr)	72.0 ± 8.1 (61–81)^a^	64.9 ± 12.7 (38–80)	66.3 ± 12.1 (38–81)
Duration of illness (yr)	7.2 ± 3.9 (1–11)	5.3 ± 3.7 (2–20)	5.6 ± 3.7 (1–20)
Previous treatments			
Transurethral resection of Hunner lesions with bladder hydrodistension (no. of patients)	6	25	31
Times on average (range)	2.6 ± 1.0 (1–3)	2.7 ± 1.2 (1–4)	2.7 ± 1.1 (1–4)
Maximum bladder capacity at the last hydrodistension (ml)	358.3 ± 120.1 (200–500)	418.3 ± 161.6 (90–700)	406.3 ± 154.2 (90–700)
DMSO intravesical instillation (no. of patients)	2	1	3
Sacral epidural anesthesia (no. of patients)	0	1	1
Medicines (no. of patients)			
NSAIDs	3	9	12
Acetaminophen	6	12	18
Opioids	4	11	15
Anticholinergic agents	1	8	9
Beta-3 adrenoceptor agonists	3	8	11
Alpha-1 adrenoceptor antagonists	3	0	3
Tricyclic antidepressant	1	7	8
SNRI	1	4	5
Pregabalin	1	6	7
Suplatast tosilate	1	3	4
PDE5 inhibitor	2	0	2
Benzodiazepine	1	0	1

DMSO = dimethyl sulfoxide; NSAIDs = nonsteroidal anti-inflammatory drugs; PDE5 = phosphodiesterase type 5; SD = standard deviation; SNRI = serotonin and noradrenaline reuptake inhibitor.

a Mean ± SD (range).

### 3.2 Treatment outcome of the low-dose PSL treatment

The mean follow-up period was 20.1 ± 14.6 mo (range, 3–62 mo). Of the 31 patients, nine discontinued PSL treatment by 12 mo due to treatment failure; however, these patients were included in the response assessment up until 12 mo. Of the remaining 22 patients, one patient requested to stop PSL treatment at 7 mo due to complete resolution of symptoms, but was included in the treatment outcome assessment up until 12 mo. A total of 21 patients continued treatment up to 12 mo at a mean PSL dose of 3.0 mg/d (range, 1.25–5.0 mg/d).

**Fig. 1:**
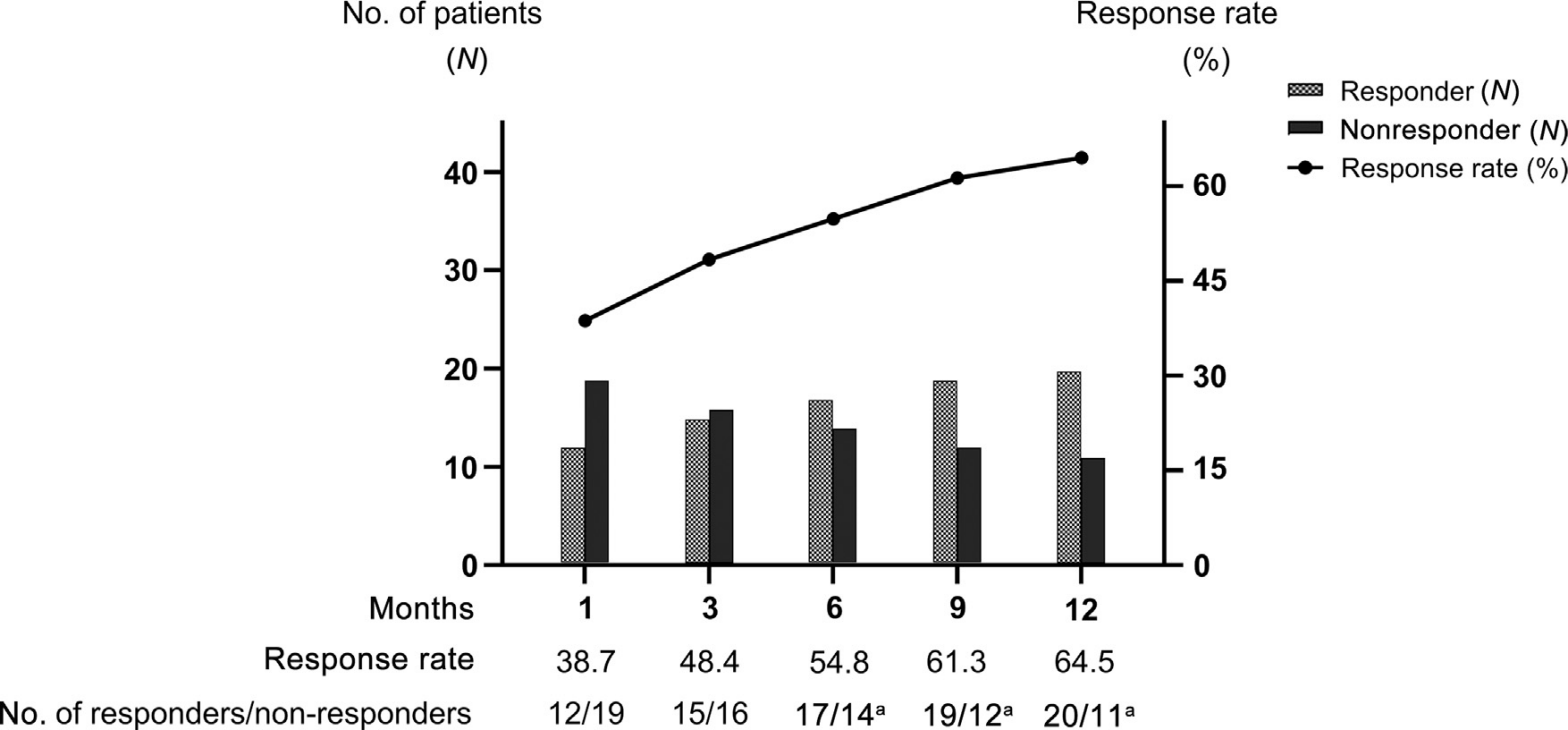
Overall response rates after PSL treatment. Treatment response was evaluated using a seven-graded global response assessment (GRA) questionnaire (markedly improved [+3], moderately improved [+2], slightly improved [+1], no change [0], slightly worse [–1], moderately worse [–2], and markedly worse [–3]). Patients who rated the efficacy as better than +2 in the GRA were defined as responders. PSL = prednisolone. ^a^ Nine patients who ceased the PSL treatment by 12 mo due to treatment failure were included as nonresponders.

**Fig. 2:**
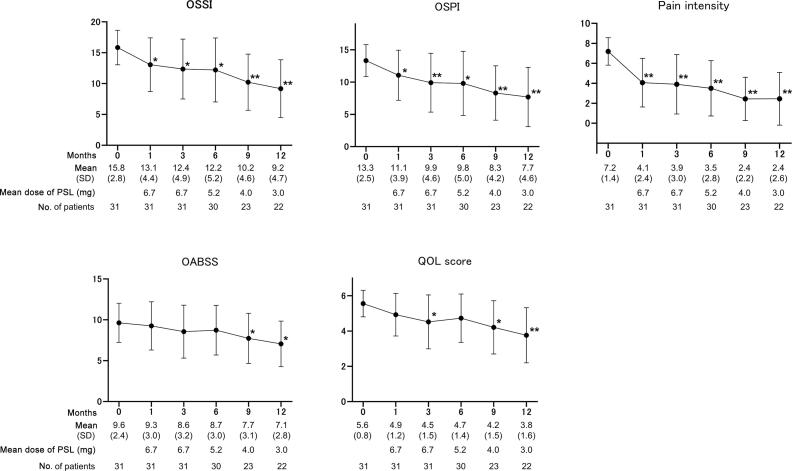
Symptom and QOL parameters during the 1-yr follow-up. OSSI/OSPI and pain intensity improved significantly from 1 mo after PSL induction, and efficacy was maintained during the follow-up period. The OABSS and QOL scores improved significantly from 9 mo after PSL induction. Values are expressed as the mean ± standard deviation (SD). Two-tailed, pairwise comparison was conducted using the Wilcoxon signed-rank test and post hoc Bonferroni correction. OABSS = overactive bladder symptom score; OSPI = O’Leary and Sant problem index; OSSI = O’Leary and Sant symptom index; PSL = prednisolone; QOL = quality of life. *p < 0.001, **p < 0.0001; statistically significant difference between each visit and baseline (0 mo).

**Fig. 3:**
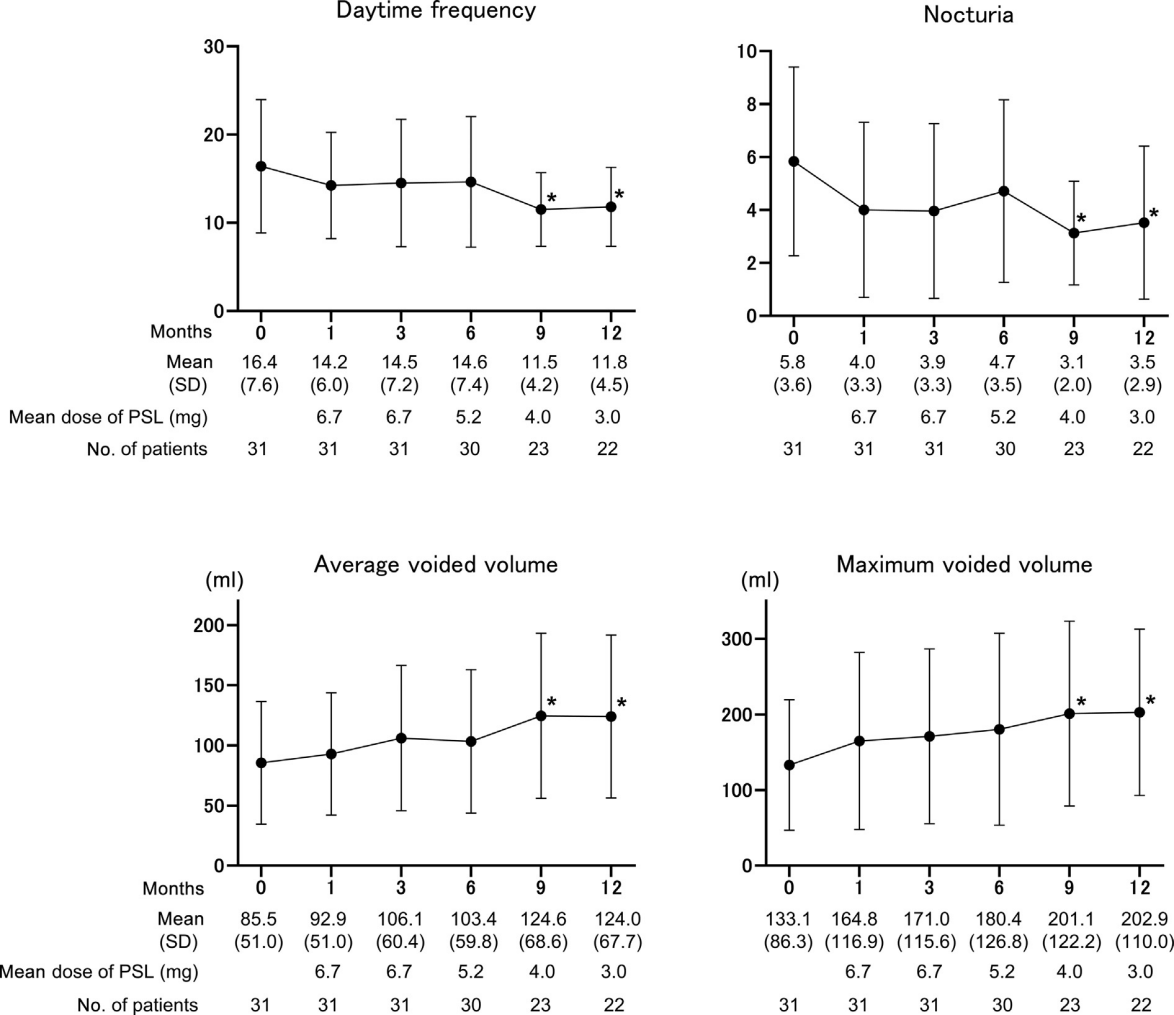
Urinary frequency and voided volume during the 1-yr follow-up. Daytime frequency and nocturia were reduced significantly from 9 mo after PSL induction, in parallel with a significant increase in average and maximum voided volume. A two-tailed, pairwise comparison was conducted using the Wilcoxon signed-rank test and post hoc Bonferroni correction. Values are expressed as the mean ± standard deviation (SD). PSL = prednisolone. *p < 0.001, **p < 0.0001; statistically significant difference between each visit and baseline (0 months).

**Fig. 4:**
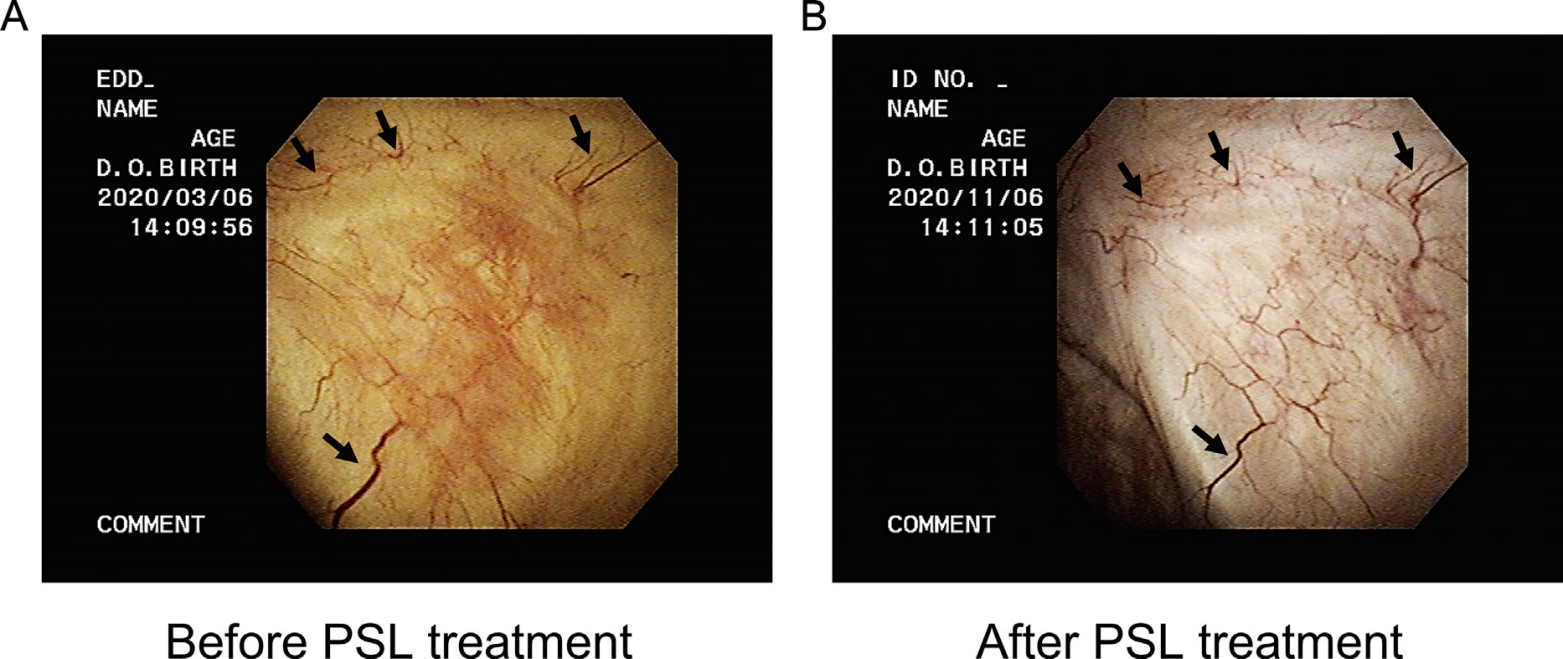
Representative cystoscopic images show disappearance of Hunner lesions after PSL treatment. A 70-yr-old male patient with HIC developed Hunner lesions at 6 mo after the second session of electrocautery of Hunner lesions and subsequent intravesical dimethyl sulfoxide injections. The Hunner lesions disappeared 8 mo after the start of PSL treatment at a daily dose of 5 mg. (A) Hunner lesions in the posterior wall, observed before the PSL treatment. (B) Disappearance of the Hunner lesions at 8 mo after the initiation of PSL treatment. The black arrows point to the same vessels seen in the left-hand image. HIC = Hunner-type interstitial cystitis; PSL = prednisolone.

The overall response rates at 1, 3, 6, 9, and 12 mo were 38.7%, 48.4%, 54.8%, 61.3%, and 64.5% at mean PSL doses of 6.7, 6.7, 5.2, 4.0, and 3.0 mg, respectively (Fig. 1). Compared with the baseline values, the OSSI/OSPI score and pain intensity fell significantly from 1 mo after PSL induction, and efficacy was maintained over the course of 1 yr (Fig. 2). The OABSS and QOL score, daytime frequency, and nocturia significantly improved from 9 mo after PSL induction, in parallel with a significant increase in average and maximum voided volume (Fig. 3). Hunner lesions disappeared after PSL treatment in four responders who underwent cystoscopy (Fig. 4). There were no pretreatment parameters predictive of treatment response at 12 mo (Supplementary Table 2).

Of the 20 responders at 12 mo (including one responder that discontinued PSL at 7 mo), eight were still continuing treatment at a maintenance dose of 1.25–2.5 mg at the end of the study (February 2023), and the remaining 12 discontinued treatment by 24 mo due to symptom remission. Of these 12 patients, five remained symptom free at the end of the study, and seven relapsed at 2 (*N* = 2), 3 (*N* = 2), 6 (*N* = 1), 26 (*N* = 1), and 28 (*N* = 1) mo after treatment cessation. Two nonresponders at 12 mo continued PSL treatment after 12 mo with a dose of 5.0–7.5 mg until treatment failure (15 and 21 mo). Patients with treatment failure immediately received salvage endoscopic surgery or intravesical DMSO treatment after PSL treatment cessation.

### 3.3 Safety of the low-dose PSL treatment

Observed adverse events are shown in Table 2. The most common adverse event was body weight gain (four patients, 12.9%), followed by hypertension and glucose intolerance (two patients, 6.5%, respectively). Hypertension and glucose intolerance were restored to the normal range by temporal oral medications. All side effects were tolerable, and no patient ceased PSL treatment due to the adverse events.

**Table 2:** Adverse events after PSL treatment

Adverse events	No. of patients (%)
Body weight gain	4 (12.9)
Hypertension	2 (6.5)
Glucose intolerance	2 (6.5)
Dizziness	1 (3.2)
Febrile urinary tract infection	1 (3.2)

PSL = prednisolone.

## 4 Discussion

Here, we observed that low-dose PSL significantly ameliorated bladder pain and voiding dysfunction, increased bladder capacity, and improved QOL for 12 mo or longer in patients with refractory HIC, without causing serious adverse events. Of note, low-dose oral PSL provided marked relief from pelvic/urethral pain, which is the hallmark and most troublesome symptom of HIC, from 1 mo after the start of treatment.

To date, Hunner lesion–targeted therapies, including local electrocautery or injection of a corticosteroid, have been regarded as cardinal treatments for HIC [1], [2]. However, the duration of the efficacy of these treatments is relatively short (≤12 mo), and most patients require repeat procedures to maintain the therapeutic effects [21], [22]. Furthermore, multiple sessions of electrocautery of Hunner lesions may affect bladder anatomy and reduce bladder capacity, potentially leading to bladder contraction [23]. Recently, we observed that bladder capacity decreased by approximately 50 ml per single session in patients with HIC who had undergone multiple sessions of electrocautery of Hunner lesions [23]. Given this possible risk of bladder malformation, electrocautery of Hunner lesions should be performed as few times as possible. Thus, alternative, conservative, durable treatments are needed to treat patients with HIC.

Based on the presence of intense chronic inflammation in the HIC bladder, prior studies examined the utility of immunomodulatory therapies for patients with HIC; these include cyclosporine A or corticosteroids. To date, cyclosporine A has been the most frequent treatment for HIC, and it improves pain and urinary symptoms in refractory patients significantly [14], [15], [16], [24], [25], [26]. However, the drug is associated with significant adverse events such as elevated serum creatinine levels, gingival hyperplasia, hypertension, and susceptibility to lymphoproliferative malignancies and skin malignancies [14], [15], [24], [25]. These serious complications mean that many urologists are unwilling to use cyclosporine A as a treatment for HIC. By contrast, corticosteroids seem to be safer, more cost effective, and familiar to many. Currently, corticosteroids are used widely to treat autoimmune or allergic disorders. However, few studies have examined the use of corticotherapy for HIC [12], [13], [27], [28], with only two studies being undertaken in the past 50 yr [12], [13]. In 2004, Hosseini et al [12] reported the results of an 8 wk course of PSL for patients with HIC, with an initial daily dose of 30 mg, decreasing by 5 mg weekly thereafter to reach a maintenance dose of 5 mg. The results showed that PSL with a mean daily dose of 6.3 mg improved OSSI/OSPI significantly in 50% of patients, with no serious adverse events. In 2005, Soucy and Gregoire [13] reported that PSL treatment, starting with a daily dose of 25 mg for at least 1–2 mo, reduced pain significantly and improved the urinary symptoms of 14 patients with refractory HIC, with an overall response rate of 65% reported over a mean follow-up period of 16 mo. In that study, however, long-term administration of a relatively high dose of PSL caused significant comorbid side effects, including two cases of new-onset diabetes type Ⅱ and two cases of pneumonia, which led ultimately to treatment cessation in three patients (21.4%). These significant complications were prevented at a lower maintenance PSL dose of 5–10 mg/d.

In the present study, we attempted to treat HIC with a low dose of PSL. Today, there are no clear indications regarding the appropriate dose of PSL for HIC. Generally, high doses of PSL (>20 mg/body) are often used initially to treat naïve/recurrent systemic autoimmune diseases [18]. However, the risk of systemic adverse events to PSL increases in a dose-dependent manner. Past evidence shows that 7.5 mg of PSL may be the threshold for increasing the risk of serious adverse events such as depression, listlessness, glaucoma, increased blood pressure, and glucocorticoid-induced osteoporosis [18], [19]. Based on this evidence, we chose to administer PSL at a daily dose of <7.5 mg/body for refractory HIC and obtained a successful outcome with no serious adverse events.

Lastly, the importance of bladder histological assessment when treating HIC with PSL should be emphasized. We administered PSL to HIC patients whose bladders showed histological chronic inflammatory changes and characteristic features of HIC. Such tailored PSL treatment based on histological evaluation might improve success rates and reduce the inappropriate use of PSL.

This study has several limitations, which include its retrospective nature and relatively small sample size, in addition to the potential placebo effect due to consistent follow-up conducted by the same urologist (Y.A.), which together limit the methodological quality. The lack of a control group also limits the interpretation of the demonstrated efficacy of PSL treatment. Further prospective, randomized, placebo-control studies of low-dose PSL treatment for HIC are warranted.

## 5 Conclusions

We observed that low-dose oral PSL significantly improved bladder pain, urinary symptoms, and QOL in patients with refractory HIC, with results comparable with those of prior observational studies of treatment with high PSL doses or cyclosporine A. The low-dose regimen may have contributed to the satisfactory safety results in the present study. Further prospective evaluation is warranted to verify the potential efficacy and safety of low-dose PSL treatment for this intractable disease.

  ***Author contributions*:** Yoshiyuki Akiyama had full access to all the data in the study and takes responsibility for the integrity of the data and the accuracy of the data analysis.

  *Study concept and design*: Akiyama.

*Acquisition of data*: Akiyama.

*Analysis and interpretation of data*: Akiyama.

*Drafting of the manuscript*: Akiyama.

*Critical revision of the manuscript for important intellectual content*: Niimi, Nomiya, Taguchi, Yamada, Sato, Yamada, Maeda, Ushiku, Kume, Homma.

*Statistical analysis*: Akiyama.

*Obtaining funding*: Akiyama.

*Administrative, technical, or material support*: Akiyama.

*Supervision*: Homma.

*Other*: None.

  ***Financial disclosures:*** Yoshiyuki Akiyama certifies that all conflicts of interest, including specific financial interests and relationships and affiliations relevant to the subject matter or materials discussed in the manuscript (eg, employment/affiliation, grants or funding, consultancies, honoraria, stock ownership or options, expert testimony, royalties, or patents filed, received, or pending), are the following: None.

  ***Funding/Support and role of the sponsor*:** This study was supported by a KAKENHI Grants-in-Aid from the Japanese Society for the Promotion of Science (JSPS; grant number 22K16788 to Yoshiyuki Akiyama), and by a Health Labour Sciences Research Grant from the Ministry of Health, Labour, and Welfare (grant number 21446301 to Yukio Homma). The sponsor played a role in the design and conduct of the study, collection, management, analysis and interpretation of the data and preparation of the manuscript.

  ***Acknowledgement:*** We thank all the participants for agreeing to take part in the study.
