# Supplementary material for: Efficacy and Safety of Low-dose Oral Prednisolone for Patients with Refractory Hunner-type Interstitial Cystitis
Source: Eur Urol Open Sci. 2023 Aug 26;56:1–8. doi: 10.1016/j.euros.2023.07.006 (PMC10562155; doi:10.1016/j.euros.2023.07.006)
Supplement: Supplementary data 1 [file mmc1.docx]

**Supplementary Table 1**. Eligibility criteria for steroid therapy

| IC/BPS symptom recurrence | |
| --- | --- |
|  | Patients with IC/BPS with Hunner lesions previously treated two times or more with electrocautery of Hunner lesions. |
|  | Recurrence after the last electrocautery of Hunner lesions with bladder hydrodistension or intravesical DMSO treatment |
|  | O’Leary-Sant Interstitial Cystitis Symptom and Problem Index scores ≥ 6 and 6, respectively |
|  | Pain intensity^†^ ≥ 4 |
|  | Global response assessment^‡^ ≤ -1 |
| Comorbid conditions | |
|  | Negative for pregnancy |
|  | Negative for Treponema pallidum, and hepatitis B, hepatitis C, and HIV virus serology |
|  | No history of genital herpes, any cancer, diabetes, hypertension, tuberculosis, invasive fungal infection, untreated cataract, glaucoma, arterial thrombosis, and human immunodeficiency disease |
| Blood test and urinary analysis | |
|  | Normal serum complete blood count, electrolytes, renal function (creatinine and blood urea nitrogen), hepatobiliary enzymes, and hemoglobin A1c. |
|  | Negative for urine culture and no symptomatic urinary tract infection |
| Histology^¶^: Evidence of chronic inflammatory changes compatible to the HIC bladder | |
|  | Presence of inflammatory infiltrates predominantly composed by lymphoplasmacytic cells outnumbering granulocytes |
|  | Presence of frequent formation of lymph follicles/aggregates in the subepithelial layer |
|  | Presence of stromal edema, fibrosis, and hyperemia |
|  | Presence of epithelial denudation |

DMSO: dimethyl sulfoxide; HIC: Hunner type interstitial cystitis; IC/BPS: interstitial cystitis/bladder pain syndrome

^†^Assessed using an 11-point pain intensity numerical rating scale from 0 (“no pain”) to 10 (“the worst pain ever”).

^‡^A 7-point symmetric scale for patients’ subjective evaluation of treatment response with markedly improved (+3), moderately improved (+2), slightly improved (+1), no change (0), slightly worse (-1), moderately worse (-2), and markedly worse (-3).

¶Modified from the reference [5].

**Supplementary Table 2.** Logistic regression analysis of baseline parameters for prediction of treatment response at 12 months

|  | **Odds Ratio** | **95% Confidence Interval** | ***P* value** |
| --- | --- | --- | --- |
| **No. (male/female)** | 0.16 | 0.002–11.14 | 0.40 |
| **Mean age (years)** | 1.01 | 0.89–1.15 | 0.80 |
| **Duration of illness (years)** | 1.02 | 0.68–1.48 | 0.91 |
| **OSSI** | 1.97 | 0.76–8.76 | 0.24 |
| **OSPI** | 0.51 | 0.12–1.45 | 0.25 |
| **Pain intensity^¶^** | 1.45 | 0.54–5.39 | 0.49 |
| **OABSS** | 0.70 | 0.23–1.64 | 0.42 |
| **QOL score^#^** | 0.57 | 0.008–11.14 | 0.75 |
| **Daytime frequency** | 0.89 | 0.59–1.20 | 0.48 |
| **Nocturia** | 0.91 | 0.43–1.89 | 0.79 |
| **Average voided volume (mL)** | 1.02 | 0.79–1.33 | 0.85 |
| **Maximum voided volume (mL)** | 1.00 | 0.87–1.14 | 0.98 |
| **Maximum bladder capacity at the last session of hydrodistension (mL)** | 0.99 | 0.97–1.00 | 0.08 |

^¶^Assessed using an 11-point pain intensity numerical rating scale from 0 (“no pain”) to 10 (“the worst pain ever”).

^#^Assessed on a 7-grade quality of life (QOL) scale derived from the International Prostate Symptom Score, with 0 indicating “excellent” and 6 indicating “terrible”.

OABSS: overactive bladder symptom score; OSSI/OSPI: O’Leary and Sant symptom index/O’Leary and Sant problem index; QOL: quality of life
